# Social Media Use, Influencer Status, and Outdoor Risk-Taking in Australian Adults: Cross-Sectional Survey

**DOI:** 10.2196/73089

**Published:** 2025-08-21

**Authors:** Samuel Cornell, Amy Peden

**Affiliations:** 1 UNSW Beach Safety Research Group, University of New South Wales Sydney, Kensington, Sydney, NSW 2033, Australia, 61 (2) 9065 3863; 2 School of Population Health, Faculty of Medicine and Health, University of New South Wales Sydney, Sydney, Australia

**Keywords:** social media, Australia, selfies, outdoors, leisure, tourism, safety

## Abstract

**Background:**

There is growing awareness of the broader health-related harms of social media; yet, research on social media–related injury mortality and morbidity remains limited. Emerging evidence suggests links between excessive social media use and increased risks of self-harm, cyberbullying-related distress, and dangerous viral challenges, but there has been limited research on the link between time spent on social media and environmental risk-taking, such as risky selfies. However, comprehensive epidemiological studies and policy-driven interventions remain scarce, highlighting the need for further investigation into the public health implications of digital engagement.

**Objective:**

This research aimed to examine the relationship among self-reported time spent on social media, influencer status, and risk-taking behaviors among Australians, considering implications for injury prevention.

**Methods:**

A cross-sectional survey of Australian social media users (N=509) was conducted using stratified quotas to approximate national distributions by age, sex, and geographical location. Participants reported their average daily time spent on social media, whether they identified as a social media influencer, and whether they had ever engaged in risk-taking behavior to create social media content. Associations between categorical variables (eg, influencer status and risk-taking) were examined using Pearson chi-square tests and supplemented with odds ratios (ORs) and 95% CIs. Independent samples 2-tailed *t* tests were used to compare mean time spent on social media between risk-takers and non–risk-takers.

**Results:**

Among participants, 48 (9.4%) self-reported engaging in risk-taking behavior in the outdoors. Influencers were significantly more likely to report risk-taking (28/58, 48.3%) compared to noninfluencers (20/451, 4.4%; *χ*²_1_=110.57, *P*<.001). Risk-takers (n=48) also spent significantly more time on social media (mean=2.05, SD 1.04) compared to non-risk-takers (n=461; mean 1.37, SD 1.04; *t*_57.22_=4.31, *P*<.001). In multivariate analyses, influencers (OR 20.11), males (OR 2.00), and younger age groups (eg, OR 33.06 for 18‐24 vs 55‐64 years) had significantly higher odds of reporting risk-taking.

**Conclusions:**

Outdoor risk-taking for content creation is associated with influencer status and greater time spent on social media. These findings suggest that policy makers should prioritize regulations addressing risky social media behaviors and hold platforms accountable for promoting harmful content. Social media platforms should implement real-time alerts, pop-up warnings, and geolocated safety information to discourage risky behaviors. Public health practitioners should engage influencers to promote safer content norms and develop targeted injury prevention strategies.

## Introduction

The rise in social media use has impacted human behavior, including outdoor activities. One notable change driven by smartphone and social media use is the increasing tendency to take photographs and selfies in risky outdoor settings [1]. While most selfies and photographs are harmless, the pursuit of visually engaging content for social media has led some individuals to take significant risks, often in hazardous locations such as cliffs, waterfalls, and other remote areas [2]. This phenomenon, fueled by social media platforms’ algorithmically driven reward systems (likes, comments, and increased visibility), has emerged as a public health concern, resulting in injuries and deaths [3,4].

Social media platforms are intentionally designed to maximize user engagement through algorithmic content curation, variable reward schedules, and metrics like likes, shares, and follower counts. These mechanisms reinforce high-engagement content, which often includes dramatic or risky imagery, indirectly incentivizing risk-taking behaviors [5,6], which we define in this study as self-reported engagement in actions in outdoor settings that could endanger personal safety, undertaken specifically for the purpose of creating content for social media platforms.

Theoretical frameworks help explain why individuals may engage in these behaviors. The uses and gratifications theory suggests that people actively choose media and content to satisfy specific psychological and social needs, such as entertainment, social interaction, and self-expression [7]. In the context of social media, this theory explains why users—especially influencers—may take risks to create engaging content that meets these needs and attracts audience attention [8].

Previous research has highlighted the link between social media-driven risk-taking and injury, particularly in the context of extreme selfies and dangerous outdoor activities. Studies suggest that social media engagement reinforces risky behaviors through social validation mechanisms [9], yet the specific role of time spent on social media and influencer status in driving these behaviors has remained underexplored.

Negative impacts of social media use on mental health and well-being are increasingly recognized. Excessive social media use has been linked to higher rates of anxiety, depression, self-harm, and exposure to cyberbullying [8,10,11]. These mental health stressors can increase sensation-seeking behavior and risk-taking, particularly among young users seeking validation or coping with digital pressures [12].

Furthermore, previous studies have shown that poor mental health outcomes such as anxiety and depression can contribute to impulsivity and reduced risk appraisal, particularly among adolescents and young adults [12]. These psychological factors may increase the likelihood of engaging in risky behaviors to seek social validation or to cope with negative affect, further compounding the harms associated with excessive social media use.

Influencers, who derive social and economic capital from online visibility, often push content boundaries to maintain engagement [11,13-15]. Their behaviors are highly visible and frequently imitated, contributing to the normalization of risk-taking in pursuit of content creation [16].

While previous studies have documented incidents of injury and death associated with risky selfies and outdoor stunts [1], none have quantitatively examined behavioral correlates such as time spent on social media or self-identified influencer status. These variables are central to understanding the extent to which social media engagement drives behavioral risk.

This study is the first to specifically investigate the relationship between time spent on social media, influencer status, and self-reported risk-taking behavior in outdoor environments. Such behaviors not only pose risks to individuals but can also burden emergency services [3], complicate park and land management, and influence the behavior of broader online audiences. Understanding these relationships is critical for developing targeted interventions that address the underlying social and psychological drivers of risky behaviors for content creation.

## Methods

### Ethical Considerations

This study involved human participants and was approved by the University of New South Wales Sydney Human Research Ethics Committee (project number HC230479). All participants provided informed consent before commencing the survey. Data were collected anonymously through an established third-party panel provider (Dynata) and were fully deidentified before analysis. No personally identifying information was accessible to the research team at any stage. Participants received standard, non–research-specific compensation in the form of points via Dynata’s rewards system. No identifiable images or personal visual data are included in this manuscript or the supplementary materials.

### Study Design and Setting

This study used a cross-sectional, self-report survey design to investigate social media use and risk-taking behaviors, which we define in this study as self-reported engagement in actions in outdoor settings that could endanger personal safety. The survey was administered online using the Dynata panel survey platform, which provided a sample that was demographically balanced through stratified quotas based on age, sex, and geographical location. Data collection occurred from October 26 to November 2, 2023.

### Participants and Recruitment

Participants were eligible if aged 18 years or older, resided in Australia, and used a social media platform. Recruitment was managed by Dynata, an established international online panel provider. Dynata uses multisource recruitment, including partnerships with websites, email lists, and mobile apps, to reach a broad and diverse participant pool. Stratified quota sampling was used to approximate the Australian population distribution by age, sex, and geographical location, including urban versus regional residence based on postal code. Geographical stratification was applied based on state or territory population distribution and urban versus regional residence based on the residential postal code (zip code) of the respondent. Participants received compensation through Dynata’s points-based reward system. The research team was not involved in recruitment or incentives. Although quotas were used to improve demographic balance, the sample was drawn from a nonprobability online panel, and no post-stratification weighting was applied. As such, results should not be interpreted as nationally representative but rather as reflecting patterns among a diverse sample of Australian social media users.

### Survey Measures

The wider survey included a combination of closed- and open-ended questions. This study focuses on 3 key items, which were asked using the following exact wording:

1. Time spent on social media: *“On average, how much time do you spend on social media each day?”* Response options: Less than 30 minutes, 30 minutes to 1 hour, 1 to 2 hours, 2 to 3 hours, more than 3 hours.
2. Influencer status: *“Do you consider yourself a social media influencer?”* Response options: Yes/No.
3. Risk-taking behavior: *“Have you ever taken risks in outdoor or recreational settings specifically to create content for social media?”* Response options: Yes/No.

Demographic variables, including age, gender, and geographical location, were also collected.

### Analysis

Categorical responses for time spent on social media (eg, “Less than 30 min”) were converted into approximate numerical values (eg, 0.5 h for “Less than 30 min”). This allowed the creation of a continuous variable to calculate means and standard deviations, which supports clearer comparisons between groups. This approach is commonly used in social science and health research where ordinal categories represent underlying continuous behaviors, and it allows for the application of parametric tests [17]. Analyses were conducted in R (version 2024.09.0+375; R Core Team) using dplyr for data manipulation, tidyr for data cleaning, and ggplot2 for visualization.

Summary statistics (means, SDs, and frequencies) were calculated for all key variables. Chi-square tests were used to assess associations between categorical variables, such as influencer status and risk-taking behavior. Independent samples 2-tailed *t* tests were used to compare mean time spent on social media between self-reported risk-takers and non–risk-takers. Odds ratios (ORs) and 95% CIs were also calculated from 2×2 contingency tables to quantify the strength of associations between risk-taking and key predictors, including influencer status, gender, and time spent on social media.

## Results

The survey had a total of 509 respondents (age: mean 45.9, SD 16.8 years). In total, 256 (50.3%) identified as female, and 11.4% (n=58) considered themselves social media influencers. Participants reported spending an average of 1.43 (SD 1.06) hours per day on social media. Among the sample, 9.43% (n=48) of them reported having taken risks to create social media content.

A significant association was also found between age and risk-taking behavior (*χ*²_5_=62.61, *P*<.001), with younger participants being more likely to report engaging in risk-taking. Compared to adults aged 55-64 years (reference group), the odds of reporting risk-taking were significantly higher among younger age groups: 18‐24 years (OR 33.06, 95% CI 6.57‐806.26; *P*<.001), 25‐34 years (OR 12.91, 95% CI 2.49‐318.16; *P*=.0007), and 35‐44 years (OR 7.31, 95% CI 1.31‐185.01; *P*=.0223). The proportion of participants engaging in risk-taking was highest among those aged 18‐24 years (32.3%) and progressively declined with age, with only 1.2%‐1.3% of individuals aged 55 and older engaging in such behavior. Gender differences in risk-taking behavior were also statistically significant (*χ*²_1_=4.30, *P*=.038). Males (12.5%) were significantly more likely to report engaging in risk-taking behavior compared to females (6.6%). A chi-square test revealed a significant association between identifying as a social media influencer and reporting risk-taking behavior (*χ*²_1_=110.57, *P*<.001). Cross-tabulation showed that 48.3% (n=28) of influencers reported risk-taking, compared to only 4.4% (n=20) of noninfluencers (Table 1). An independent samples 2-tailed *t* test demonstrated that participants who reported risk-taking behavior spent significantly more time on social media (mean 2.05, SD 1.04) compared to those who did not report risk-taking (mean 1.37, SD 1.04; *t*_57.22_=4.31, *P*<.001).

**Table 1.** Chi-square and *t* test analysis of risk-taking behavior by age, gender, influencer status, and social media usage.

Variable and category	Risk-taking, No, n (%)	Risk-taking, Yes, n (%)	Chi-square (*df*; *P* value)
Age group (years)			62.607 (5; <.001)
18‐24	44 (67.7)	21 (32.3)	
25‐34	76 (84.4)	14 (15.6)	
35‐44	87 (90.6)	9 (9.4)	
45‐54	90 (97.8)	2 (2.2)	
55‐64	80 (98.7)	1 (1.3)	
≥65	84 (98.8)	1 (1.2)	
Gender			4.300 (1; .038)
Male	218 (87.5)	31 (12.5)	
Female	239 (93.4)	17 (6.6)	
Other or prefer not to say	4 (100.0)	0 (0.0)	
Influencer status			110.574 (1; <.001)
Yes (influencer)	30 (51.7)	28 (48.3)	
No (noninfluencer)	431 (95.6)	20 (4.4)	
Average daily time spent on social media			23.201 (4; <.001)
<30 min	99 (99.0)	1 (1.0)	
30 min-1 h	135 (92.5)	11 (7.5)	
1‐2 h	118 (91.5)	11 (8.5)	
2‐3 h	53 (79.1)	14 (20.9)	
>3 h	56 (83.5)	11 (16.4)	

In bivariate analyses, influencer status, male gender, younger age, and higher daily social media use were all significantly associated with increased odds of reporting risk-taking behavior (Table 2). Influencers had 20.11 times the odds of reporting risk-taking compared to noninfluencers (95% CI 10.16‐39.81, *P*<.001). Males had 2 times the odds of reporting risk-taking compared to females (95% CI 1.04‐3.85, *P*=.033). Compared to adults aged 55-64 years (reference group), the odds of reporting risk-taking were significantly higher among younger age groups: 18‐24 years (OR 33.06, 95% CI 6.57‐806.26; *P*<.001), 25‐34 years (OR 12.91, 95% CI 2.49‐318.16; *P*=.0007), and 35-44 years (OR 7.31, 95% CI 1.31‐185.01; *P*=.0223). No significant differences were observed for the 45- to 54-year or 65- to 99-year age groups. Individuals spending more than 1 hour per day on social media had 3.09 times the odds of reporting risk-taking compared to those spending 1 hour or less per day (95% CI 1.57‐6.09, *P*=.001).

**Table 2.** Odds ratios (ORs), 95% CIs, and *P* values for reporting risk-taking behavior by influencer status, gender, age, and daily social media use. Reference categories: non-influencer, female, 55‐ to 64-year age group, and ≤1 hour/day social media use. “Ref” denotes the reference group.

Predictor and comparison	OR	95% CI	*P* value
Influencer status: influencer vs noninfluencer (ref)	20.11	10.16‐39.81	<.001
Gender: male vs female (ref)	2.00	1.04‐3.85	.033
Daily social media use: >1 hour/day vs ≤1 hour/day (ref)	3.09	1.57‐6.09	.001
Age group (years)			
18‐24 vs 55‐64 (ref)	33.06	6.57‐806.26	<.001
25‐34 vs 55‐64 (ref)	12.91	2.49‐318.16	.0007
35‐44 vs 55‐64 (ref)	7.31	1.31‐185.01	.0223
45‐54 vs 55‐64 (ref)	1.67	0.13‐53.05	1.000
65‐99 vs 55‐64 (ref)	0.95	0.02‐37.58	1.000

## Discussion

### Principal Findings

This study provides important insights into the associations between social media use, influencer status, and risk-taking behaviors, with implications for injury prevention strategies in outdoor settings. The strongest statistical finding shows that risk-taking behavior for social media is significantly more prevalent among younger individuals, particularly those aged 18‐24, who had over 30 times the odds of reporting risk-taking compared to adults aged 55‐64. Adults aged 25-34 years and those aged 35-44 years also had significantly increased odds of risk-taking. These findings align with existing literature on developmental psychology, where younger adults exhibit greater sensation-seeking tendencies and risk-taking behavior [12,18]. Public health interventions should therefore focus on younger demographics, where risk perception and decision-making processes are still developing.

Social media influencers and individuals who self-reported greater average daily time on social media were also significantly more likely to engage in risk-taking behaviors for content creation. Such behaviors, which can include climbing cliffs [1], entering unsafe waters, or ignoring safety warnings, may elevate the risk of injury or death when in the outdoors [3].

Gender differences in risk-taking were also observed. Males had twice the odds of reporting risk-taking compared to females. This finding is consistent with previous research indicating that men are more inclined toward risk-taking, particularly in outdoor and physically demanding contexts [19]. Future research should further explore the interaction between gender and social media use, including whether platform dynamics amplify existing gender-based differences in risk propensity.

Social media has been characterized as addictive, with research suggesting that its design leverages psychological mechanisms such as variable reward schedules, social validation, and engagement-driven algorithms to encourage prolonged use [20] as well as platform business models strategically designed to amplify user engagement, thereby encouraging prolonged and frequent use [21]. The link between time spent on social media and changes in behavior has been well researched in the domains of well-being, mental health, and anxiety [9,10,22]. Studies often find no strong relationship and inconclusive results [10].

On the contrary, our findings indicate a strong association between social media use and risk-taking behaviors in outdoor settings. The association raises the possibility that increased social media use may influence users’ willingness to take risks, although causality cannot be determined from cross-sectional data.

Our study is novel in that it directly asks social media users to self-assess whether they engage in risk-taking behaviors in outdoor settings. The perception of one’s behavior as risky may be influenced by societal norms and the extent to which individuals believe others perceive their actions as hazardous [23]. In contrast, previous studies on social media impacts have primarily focused on subjective well-being, a construct that may be less dependent on external perceptions and more intrinsically determined [24,25].

Influencer status was also strongly associated with increased risk-taking behavior. In this study, individuals who identified as influencers had over 20 times the odds of reporting risk-taking compared to noninfluencers. Influencers often set trends that prioritize visually striking or extreme content, which may prompt followers to imitate risky behaviors in pursuit of attention, validation, or perceived status [4].

These trends have significant implications for injury prevention efforts. Social media plays a critical role in shaping peer norms and pressures, particularly among young adults and impressionable users [26,27]. Addressing this issue requires interventions that directly target the influence of social media on behavioral norms; perhaps also the influence of the influencer.

Given the strong association between time spent on social media and risk-taking behavior, public health interventions should consider targeting excessive social media use as a potential risk factor but also advocate for the removal of content that glorifies risk-taking behaviors. Addressing compulsive social media engagement through media literacy initiatives may contribute to reducing outdoor risk-taking. Future research is needed to establish whether social media exposure directly increases risk-taking or if individuals predisposed to risk-seeking behaviors are more likely to engage with social media. Platforms have a responsibility to collaborate with safety organizations and influencers to encourage the creation and dissemination of responsible content. Public health practitioners should seek to collaborate with influencers who have sway over a section of the population that they are trying to reach. These efforts are crucial for breaking the cycle of risky behaviors perpetuated by social media and preventing avoidable injuries.

These findings align with established public health frameworks that emphasize modifying environmental, social, and individual-level risk factors. For example, the Haddon Matrix [28], widely used in injury prevention, highlights the importance of addressing pre-event, event, and postevent factors, which could inform digital safety interventions. Similarly, behavior change theories such as the theory of planned behavior [29] suggest that modifying perceived norms and enhancing self-efficacy are key strategies to reduce risky behaviors online. Applying these frameworks can help guide multifaceted interventions involving policy changes, platform design modifications, and targeted education campaigns.

Several potential confounding factors should be considered when interpreting these associations. Individual traits such as sensation-seeking [12], impulsivity, and susceptibility to peer influence [16] may predispose individuals both to greater social media engagement and to risk-taking behaviors. Other factors, including socioeconomic status [18], education level, and previous experiences with outdoor activities, could also influence both social media use patterns and risk-taking propensity. These confounders should be addressed in future longitudinal or multivariate studies.

While this study highlights potential interventions—such as platform-based warnings, real-time alerts, and collaboration with influencers—it does not offer a detailed implementation or evaluation framework. This reflects the exploratory nature of the work, which aimed to establish baseline associations rather than test interventions. Further research is needed to co-design, implement, and evaluate specific harm-reduction strategies with stakeholders, including social media platforms, public health agencies, and tourism authorities. Doing so will be critical for translating these findings into actionable outcomes. The findings, and other work being conducted in this area, suggest that a public health approach is necessary to consider these types of behaviors and resulting incidents as a widespread issue that is having repercussions on a population-level basis.

Potential ethical considerations must also be acknowledged when designing interventions aimed at reducing risk-taking behaviors [30]. Efforts to remove or limit harmful online content could raise concerns about censorship, freedom of expression, and the overreach of platform policies. Balancing safety with the rights of users to share content is a complex challenge. There may need to be more discussion between ethicists, legal scholars, public health academics, and the public to determine what people are willing to lose for the sake of increased online safety.

These findings suggest the need for policy makers to consider regulations or guidelines addressing risky online behaviors, including the promotion of hazardous content. Social media platforms should be encouraged or required to implement design changes such as pop-up warnings, geolocation-based risk alerts, and clearer community guidelines discouraging risk-taking behaviors.

### Limitations

A key limitation of this study is its cross-sectional design, which restricts the ability to draw causal inferences. While significant associations were found between time spent on social media, influencer status, and self-reported risk-taking behaviors, the direction of these relationships cannot be determined. It remains unclear whether increased social media use contributes to greater risk-taking in outdoor settings or whether individuals predisposed to risk-taking are more likely to engage with social media platforms and content. Longitudinal or experimental research designs are needed to establish causal pathways and better understand the mechanisms driving these behaviors. Nevertheless, our study reveals that risk-takers spend significantly more time on social media. Although quota sampling was used to improve demographic balance, the sample was drawn from a nonprobability online panel and no statistical corrections were applied to adjust for sampling bias. These factors limit the generalizability of the findings, which should be interpreted with caution.

This study did not examine broader contextual or psychosocial factors that may influence risk-taking behavior in the social media environment, such as peer influence, personality traits, or platform-specific dynamics. Future research should consider these variables to better understand the mechanisms driving risky content creation. Finally, while ORs provided insights into demographic and behavioral predictors, some confidence intervals were wide, reflecting small numbers of risk-takers in certain subgroups. This should be considered when interpreting effect sizes.

### Conclusions

This study offers a brief overview of the role of time spent on social media in shaping risk-taking in the outdoors and its broader implications for public health. The study determined a significant link between time spent on social media, influencer status, and risk-taking. Time spent on social media may need to be considered by public health practitioners and researchers when designing or testing interventions to improve safety in the outdoors at places frequented by social media users. Future research is critical to explore causal pathways between social media use and injury risks, as well as platform-specific trends that influence behavior. Policy makers should prioritize regulations addressing risky online behaviors and encourage platform accountability. Social media companies should also be engaged to co-design technological solutions aimed at discouraging risky content creation and promoting safer digital engagement.

## Abbreviations

OR: odds ratio
